# Investigation Into the Effect of Vaccination on Pulmonary Involvements in Patients With COVID‐19 Infection, Based on High‐Resolution CT Imaging

**DOI:** 10.1111/crj.70127

**Published:** 2025-09-18

**Authors:** Elahe Amani, Bentolhoda Otroshi, Mohsen Tabatabaie, Azam Moslemi, Shiva Shabani

**Affiliations:** ^1^ Dept. of Radiology, School of Medicine Arak University of Medical Sciences Arak Iran; ^2^ Clinical Research Development Unit, Ayatollah‐Khansari Hospital Arak University of Medical Sciences Arak Iran; ^3^ Health Information Management, Office of Vice Chancellor for Research Arak University of Medical Sciences Arak Iran; ^4^ Dept. of Biostatistics, School of Medicine Arak University of Medical Sciences Arak Iran; ^5^ Dept. of Infectious Diseases, School of Medicine Arak University of Medical Sciences Arak Iran

**Keywords:** Computed tomography, COVID‐19 infection, Pulmonary involvements, SARS‐CoV‐2, Vaccination

## Abstract

**Background:**

COVID‐19 infection has been a major pandemic of this century, causing deaths, economic hardship, and poverty worldwide. At the moment, vaccination remains the most effective measure against this health challenge. This retrospective study assessed the efficacy of COVID‐19 vaccination in the affected patients who had been shown positive previously by polymerase chain reaction (PCR) tests.

**Methods:**

We compared the retrospective records of 547 patients with COVID‐19 over the prior 18 months (Mar. 2021 to Sept. 2022). Data from the patients' hospital records were divided into two groups of vaccinated (*N* = 334) versus non‐vaccinated (*N* = 213) from individuals who had a prior positive PCR test. Subsequently, the patients' chest computed tomography (CT) images were evaluated and scored for the lung involvements based on a pathologically established scoring system.

**Results:**

Based on the CT image scores, it was evident that the vaccination significantly reduced the lung involvements in these patients. The severity of lung involvements was significantly less in the vaccinated than in the non‐vaccinated group, regardless of being younger or older than 65 years old. Also, the arterial oxygen saturation was significantly greater in the vaccinated than in the non‐vaccinated patients. Lastly, the vaccination had a significant effect on lowering the mortality rate and intubation in patients older than 65 years. However, there was no significant difference between the vaccinated versus the non‐vaccinated groups with respect to their admission into the ICU at the local hospital.

**Conclusions:**

Based on the results, COVID‐19 vaccination reduced the severity of lung involvements in patients significantly. Hence, it can be considered a protective measure in reducing the disease burden.

## Graphical abstract

Patients with COVID‐19 pneumonia: Vaccinated versus non‐vaccinated.

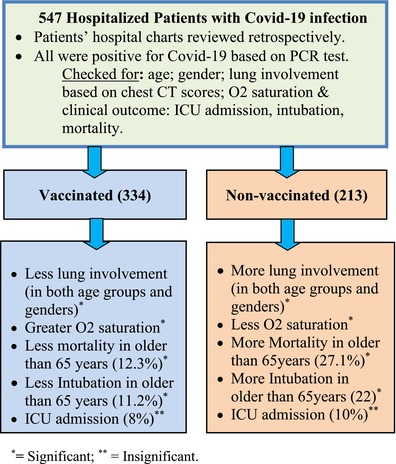

## 1 Introduction

The COVID‐19 infection is caused by the SARS‐CoV‐2 virus and has been the most challenging epidemic disease of the current century [1]. The sudden spread of this infection moved the World Health Organization (WHO) to declare it a major epidemic on March 12, 2020 [2]. Since the beginning of the COVID pandemic, concerns have emerged about the origin of the virus. Theories such as lab leakage or the possibility of bioterrorism have been proposed. Although most of these theories have not been proved, according to several researches, some doubts still remain. Therefore, a thoughtful and guided approach to biosafety research is needed to meet the associated challenges [3, 4].

To date, this infection has caused numerous deaths and much economic hardship in many nations worldwide. Although much progress has been made with respect to the clinical research and understanding the mechanism of SARS‐CoV‐2 action, many nations are still seriously challenged with this viral infection [5]. In Iran, the COVID‐19 epidemic was officially declared after the first death caused by this infection on February 18, 2020, in Qum. Soon after, this infection spread to many other provinces of the country [6], and the fifth wave of the epidemic occurred in August 2021, marking the most challenging period of this event in Iran [7].

Based on the available evidence, people at any age are at risk of getting infected with this virus; however, there are factors that put the infected individuals at higher risk. These factors include persons aged 60 years or older; people with cardiovascular, pulmonary and/or kidney diseases, diabetes, obesity, and cancer; patients with bone marrow transplants; and smokers [5]. A serious problem is that patients with COVID infection and its symptoms are indeed carriers of the virus. The most prevalent manifestations are fever, coughs, and shortness of breath [8]. Also, gastrointestinal symptoms, such as low or loss of appetite, nausea, vomiting, diarrhea, and abdominal pain, may be present in these patients without having major respiratory symptoms [9]. Notably, lung involvement has been the most common and the presence of severe pneumonia has been considered a relevant prognostic pathology [10, 11].

It is well known that the COVID‐19 virus enters the epithelial cells in the lungs' alveoli by binding to the receptors for angiotensin converting enzyme‐2 (ACE2), subsequently leading to severe pneumonia [12]. The major radiographic signs observed in the lungs' computed tomography (CT) images in these patients include opacities and ground glass lesions throughout the involved lung lobes [8]. Although a positive polymerase chain reaction (PCR) test is currently the standard diagnostic indicator of COVID‐19 infection, its low sensitivity and time‐consuming process limit the rapid screening of infected individuals [13]. Besides PCR, an alternative approach to screening infected individuals is to examine the lungs' CT images before making the diagnosis with certainty. For this purpose, high‐resolution CT (HRCT) images of the lungs are highly sensitive (> 95.5%) and reliable for evaluating the lung lesions in these patients [14]. This radiographic method has become even more desirable when combined with an artificial intelligence approach to provide a rapid automatic diagnosis [13]. In this context, the HRCT method appears to be quite capable of contributing to the diagnosis of lung lesions in patients with COVID‐19 pneumonia [15].

Currently, various therapeutic options are being applied based on their efficacy and specificity in the management of COVID‐19 pneumonia. These include the use of antiviral and anti‐inflammatory drugs, monoclonal antibodies against the virus, and a number of modulators of the human immune system. These treatments have been authorized for emergency purposes while they are being evaluated by the US Food and Drug Administration (FDA) [16]. Upon mutations in the COVID‐19 virus, the clinical outlook and the treatment approach may change, as scientists continue to search for the most effective strategies toward the management of this infection [5]. Despite the fact that physical distancing lowers the virus transmission, this measure alone may not provide complete immunity against the virus [17]. Currently, various vaccines are being developed or have already been produced.

According to CDC statements, results from clinical trials have shown that COVID‐19 vaccines are safe and effective, especially against severe illness, hospitalization, and death [18]. However, large differences in the approaches taken by different countries such as the date when countries started national vaccination campaigns, the rate of vaccinations over time, and differences in data published by official sources exist [19]. Also, before judgment about realistic vaccine effects, several issues, such as short immunity timeline after vaccination and limitations in prevention of host‐to‐host transmission [20], should be considered.

Currently, none of the COVID‐19 vaccines developed has proven to offer 100% efficacy. Hence, a fraction of the population still remains susceptible to this virus even if they have received full vaccination [21, 22]. In this context, the WHO strongly recommends that all vaccinations against COVID‐19 should follow established guidelines [23]. In the scientific community, it is believed that unless an effective and universal vaccination approach is developed, enforced, and implemented, the health of people in any part of the world cannot be guaranteed as it was the case before this epidemic [17]. Currently, results from the clinical evaluations of the various COVID‐19 vaccines have provided evidence that they are adequately effective against moderate to severe forms of this infection [24, 25, 26]. Several side effects have been reported after vaccination; most of them were mild and nonsignificant, but some serious adverse events were also reported in clinical trials leading to changing strategies. For instance, life‐threatening blood clots following vaccination with the Johnson & Johnson and the AstraZeneca vaccines have been reported, and even AstraZeneca was withdrawn by several European countries [27, 28, 29]. Finally, it should be considered that the sudden emergence of the virus, lack of adequate data about its origin, and emergency use authorization of vaccines may play a role in the lack of transparency and some hesitancy among the public [30].

### 1.1 Aim of the Study

The aim of the current study was to evaluate the efficacy of COVID‐19 vaccination in preventing pulmonary involvements and mortality rates among Iranian patients with this challenging viral infection.

## 2 Materials and Methods

### 2.1 Patient Data

Retrospective patients' data were collected and reviewed from the Medical Records Department of Amir Al‐momenin Hospital in Arak, Iran, between March 21, 2021, and September 22, 2022, based on all of the following inclusion criteria:

### 2.2 Inclusion Criteria

The criteria for including patients in this study were as follows:

- Patients visited the hospital between March 21, 2021, and September 22, 2022.
- A signed consent form from each patient was available in the hospital's medical records.
- Positive evidence of a polymerase chain reaction (PCR) test specific for COVID‐19 infection.
- A confirmed clinical diagnosis of pneumonia due to COVID‐19 infection was on record.
- Data on HRCT images of each patient's lungs were available, conducted between the third and eighth days from the onset of COVID‐19 pneumonia.
- Demographic and clinical data were available in the patients' charts, such as age, gender, evidence of COVID‐19 vaccination and dosage, clinical signs, and symptoms of pneumonia upon admission to the hospital.

### 2.3 Exclusion Criteria

The presence of any co‐morbidity or background disease in the patients' records, such as chronic lungs, cardiovascular, and kidney diseases; cancer; pregnancy; and addictions was the cause for exclusion from this retrospective study.

### 2.4 Records of CT Images

This study used a systematic examination of the patients' CT images from their lungs, providing that each CT image had been checked for at least 138 lung slices scanned in the supine position. Also, the helical images should have been recorded at the end of inspiration in the craniocaudal axis, covering an area from the thyroid glands to the superior poles of the patients' kidneys. Specifically, the following image parameters were required: 110 KVP, 50 mAs, rotation, 1.5 mm thickness, pitch 1.5, and time: 0.6 s. All of the CT images had been taken on a Siemens AG unit (Forchheim, Germany) at the Radiology Department of Amir Al‐momenin Hospital in Arak, Iran.

### 2.5 Impartiality

The analyses and diagnosis of the CT images had been conducted previously by board certified radiologists at the same hospital. To eliminate the potential for bias, each patient had been assigned an identity code. The radiologists had no prior knowledge of the patients' clinical signs and symptoms; thus, they made their diagnoses solely based on the examination of the selected CT images from the patients' lungs. In each of the five lung lobes, the radiologists searched for the following signs: *ground glass opacity*, *consolidation*, *nodules*, *pleural effusion*, *fibrosis*, and/or *mediastinal lymphadenopathy*. Then, they scored each lung lesion on a scale of zero to five. Next, they totaled the scores and filed them under each patient's code. The total score definitions for the lung involvements were as follows: 0 = 0%, 1 = 5%, 2 = 5%–24%, 3 = 25%–49%, 4 = 50%–74%, and 5 = > 75% involvements.

### 2.6 Patients Selection and Grouping

The following requirements were applied for selecting the study patients:

- They were hospitalized for at least 1 day between March 21, 2021, and September 22, 2022.
- Treatment strategies during hospital stay were based on the WHO guidelines [31] depending on the severity of the disease and available drugs as mentioned in Table 1.
- Mentioned drugs are based on hospital COVID managing protocols. To decrease confounding effects, patients with severe underlying disease, who might have had limitations in receiving specific drugs or might have needed further drugs, were not enrolled in the evaluation (as mentioned in exclusion criteria).
- Patients in the preadmission period had been only administered symptomatic treatments, such as analgesics, nasal decongestants, bromhexine syrup, and diphenhydramine.

**TABLE 1:** Prescribed drugs for hospitalized patients according to disease severity.

Mild to moderate	Remdesivir 200 mg on Day 1 followed by 100 mg on Days 2 and 3.
Severe	Remdesivir 200 mg on Day 1 followed by 100 mg on Days 2 and 3. Corticosteroids (dexamethasone 6 mg/day for 10 days or until discharge) Single IV dose of *Tocilizumab* (8 mg/kg)
Critically ill	Remdesivir 200 mg on Day 1 followed by 100 mg on Days 2 and 3. Corticosteroids (dexamethasone 6 mg/day for 10 days or until discharge) Single IV dose of *Tocilizumab* (8 mg/kg).

Once the above requirements were met, the carefully selected patients (*n* = 547) were divided into two groups of vaccinated (*n* = 334) and non‐vaccinated (*n* = 213) individuals.

### 2.7 Vaccine Administration

Patients in the vaccinated group had received one of the following vaccine types: *Astra Zeneca*, *Sinopharm*, or *COVIran Barakat*. The data from these patients were further tabulated depending on the number of vaccine doses they had received as follows:

Partial vaccination (1 dose); complete vaccination (2 or more doses; dependent on the type of vaccine), and indeterminate (time elapsed between COVID‐19 and vaccination was less than 2 weeks). The time from the onset of pneumonia to the last vaccine doses was between 14 days and 6 months in the completely and partially vaccinated group, while it was less than 14 days in the indeterminate group. Also, the dates of vaccination were compared and contrasted among the three patient groups, versus the vaccine dosage [1, 2, 3, or] they had received, regardless of the vaccine type.

### 2.8 Age and Gender Grouping

Given the likelihood that more severe lung involvements might have occurred in patients older than 65 years, the data were subdivided into two groups of less than or above 65 years of age. Then, the lung involvements in each group were compared versus being vaccinated or non‐vaccinated. Also, considering the known differences in the quality of immunity versus gender, the data for men and women were analyzed separately then subdivided for being either vaccinated or not vaccinated. Lastly, the severity of clinical signs and symptoms, such as fever, respiratory distress, abdominal pain, vomiting, nausea, diarrhea, headaches, vertigo, and seizure, was compared and contrasted among the vaccinated and non‐vaccinated groups.

### 2.9 Data Analyses

The study data were statistically analyzed on the SPSS software, version 2.0. The normality of data distribution was assessed by Shapiro–Wilk's test. Further, the data were analyzed using one‐way ANOVA, Kruskal–Wallis, chi‐square, and independent *t‐*tests. The findings were categorized and reported in six tables, reflecting the means ± standard deviations, and mean ranks, with the statistical significance level set at *p* ≤ 0.05.

## 3 Results

This study included 547 retrospective cases, divided into two groups of vaccinated (*n* = 334; 61%) versus non‐vaccinated (*n* = 213; 39%), and based on the inclusion and exclusion criteria. The patients' ages ranged from 19 to 100 years old, with the mean age being 58.7 ± 19.31 years. The mean age in the non‐vaccinated group was 52.87 ± 18.91 years, while in the vaccinated group, it was 62.36 ± 18.69 years. The selected patients for the study had stayed at the hospital at minimum for 1 day and at maximum for 31 days. The mean hospital stay in the non‐vaccinated group was 6 days, while it was 4.6 days in the vaccinated group.

In the non‐vaccinated group, the number of patients older than 65 years was 154 (46%), while those below 65 years old were 180 (54%) individuals. In the vaccinated group, there were 203 (60%) male and 132 (40%) female, while in the non‐vaccinated group, there were 207 (51%) male and 102 (49%) female.

**FIGURE 1:**
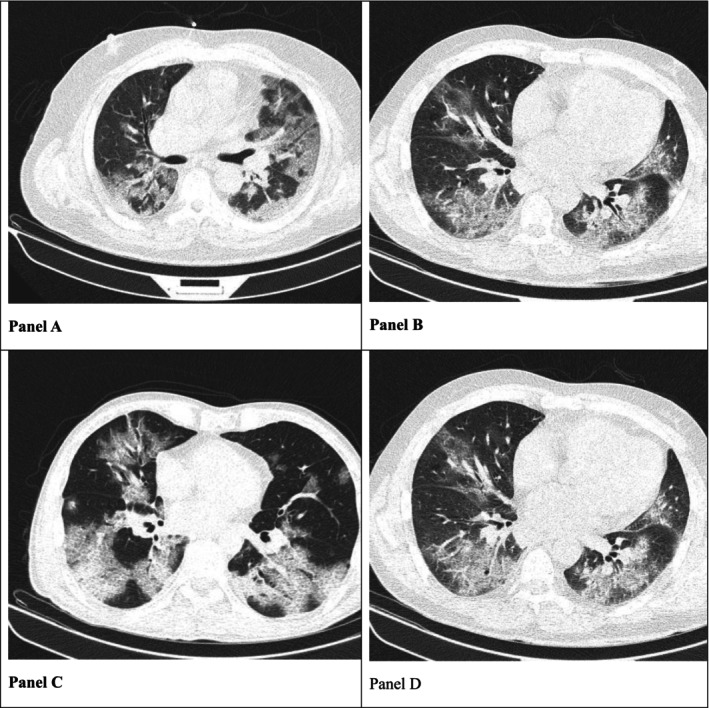
The CT images of four different patients with pulmonary involvements. Descriptions of panels: (A) non‐vaccination; (B) indeterminate vaccination; (C) partial vaccination; (D) complete vaccination. All of the four images show mainly ground‐glass opacities with peripheral and peribronchovascular distributions.

The analyzed data demonstrated that the mean severity score for lung involvements in the vaccinated patients (8.24 ± 6.11) was significantly less than that in the non‐vaccinated group (12.6 ± 6.47; *p* = 0.0001). Figure 1 presents slices of CT images from four patients with different vaccination statuses. Notably, there were no major differences with respect to their lung involvement types.

The vaccination had no significant effects on the intubation rates among the vaccinated patients (*p* = 0.24). Also, the frequency of these patients' admission to the ICU (*p* = 0.46) was not significantly different compared with those not vaccinated. Also, vaccination did not significantly reduce the mortality rate (*p* = 0.09) in the vaccinated versus non‐vaccinated patients. On the other hand, the vaccination was associated with a significant increase in the mean blood oxygen saturation in the vaccinated patients (86.44 ± 7.47), compared with those not vaccinated (84.93 ± 8.04; *p* = 0.01) (see Table 2).

**TABLE 2:** Effects of Covid‐19 vaccination on the clinical outcomes.

Clinical outcome	Vaccinated	Non‐vaccinated	*p*
Lung involvement*	8.24 ± 6.11	12.60 ± 6.47	< 0.0001
Intubation rate	8.10%	10.80%	0.24
Death rate	9.10%	13.20%	0.09
ICU admission	8.30%	10%	0.46
Oxygen saturation	86.44 ± 7.47%	84.93 ± 8.40%	< 0.01

* Lung involvement scores, ranging 0 to 25.

Further analysis of vaccination impact on the mortality and intubation rates among the patients older than 65 years showed a significant decline in the mortality (*p* = 0.007) and intubation rates (*p* = 0.03) among the vaccinated patients versus non‐vaccinated patients (see Table 3).

**TABLE 3:** Effects of Covid‐19 vaccination on the mortality and intubation rate in patients older than 65 years.

Clinical outcome	Vaccinated	Non‐vaccinated	*p*
Intubation rate	11.2	22	0.03
Death rate	12.3	27.1	0.007

There were significant differences in the severity of the lung involvements among the three vaccination groups, i.e., *partial* (5.89 ± 8.92), *complete* (5.84 ± 5.61), and *indeterminate* (7.27 ± 10.73) (*p* = 0.0001). Of note, the mean lung involvement found in the indeterminate group scored significantly higher than those with complete vaccination. Also, there were no differences in the mortality rates between the two vaccinated groups, that is, complete (8.7%) and partial (8.7%) with respect to the rates of intubation or admission to ICU (*p* = 0.42). Of note, no mortality happened in the indeterminate group. There were insignificant differences (*p* = 0.2) in the blood oxygen saturation among the patients with different vaccination types, that is, complete (87.57 ± 6.35), partial (86.40 ± 7.88), and indeterminate (87.31 ± 4.92) (see Table 4).

**TABLE 4:** Effects of vaccination on the patients clinical outcomes.

Clinical outcome	Partial vaccination	Full vaccination	Indeterminate	*p*
Lung involvement*	8.92 ± 5.89	5.61 ± 5.84	10.73 ± 7.27	< 0.0001
Intubation rate	8.7%	8.7%	0	0.4
Death rate	9.7%	8.7%	0	0.42
ICU admission	8.7%	8.7%	0	0.47
Oxygen saturation	86.40 ± 7.88	87.57 ± 6.35	87.31 ± 4.92	0.2

* Lung involvement scores, ranging 0 to 25.

The study data indicated that no significant differences were found in the frequency of clinical findings such as fever, respiratory distress, coughs, GI symptoms (abdominal pain, nausea, vomiting, diarrhea), or CNS symptoms (seizures, headaches, vertigo) in these patients, regardless of their vaccination type or status. The percentage of patients with fever and those with respiratory, GI, and CNS symptoms in the vaccinated groups were as follows: 39.8%, 91.4%, 35.3%, and 31.5%. While the corresponding percentages in the not‐vaccinated group were 36.4%, 91.2%, 36.0%, and 31.2%. The *p*‐values for the four symptom groups were 0.94, 0.92, 0.85, and 0.39, respectively (see Table 5).

**TABLE 5:** Effects of vaccination on the clinical symptoms in Covid‐19 pneumonia.

Clinical symptom	Vaccinated	Non‐vaccinated	*p*
Fever	39.8%	36.4%	0.387
Respiratory symptoms	91.4%	91.2%	0.917
Gastrointestinal symptoms	35.3%	36.0%	0.849
CNS symptoms	31.5%	31.2%	0.939

The severity of lung involvements was significantly less in the vaccinated (7.61 ± 6.02) versus the non‐vaccinated men (11.22 ± 7.04). Similarly, the mean lung involvement severity in the vaccinated women was significantly less (9.21 ± 6.12) than those who were not vaccinated (14.02 ± 5.51) (*p* = 0.0). The study findings further indicated that the vaccination significantly lowered the intensity of the lung involvements whether the patients were below or above 65 years old ((7.19 ± 5.96 Vs 9.14 ± 6.09), as compared with those who were not vaccinated (*p* = 0.0) (see Tables 6 and 7).

**TABLE 6:** Gender‐based effects of vaccination on lung involvement in COVID 19 pneumonia.

Gender	Vaccination history	Patients number	Lung involvement (mean ± SD)*	*p*
Male	No	108	11.22 ± 7.04	<0.0001
	Yes	203	7.61 ± 6.02	
Female	No	105	14.02 ± 5.51	<0.0001
	Yes	131	9.21 ± 6.12	

* Lung involvement scores, ranging 0 to 25.

**TABLE 7:** Age‐based effects of vaccination on lung involvement in COVID 19 pneumonia.

Age	Vaccination history	Patient number	Lung involvement (mean ± SD)*	*p*
≤ 65	No	161	12.79 ± 6.21	< 0.0001
	Yes	180	9.14 ± 6.09	
≥ 65	No	152	12.02 ± 7.24	< 0.0001
	Yes	154	7.19 ± 5.96	

* = Lung involvement scores, ranging 0 to 25.

We also evaluated the lung involvements in patients with different hospital admission timelines, that is, March 2021 versus September 2022. Significantly less severe lung involvements were detected in both the vaccinated and non‐vaccinated groups who were admitted late in 2022 (late timeline) compared with those admitted early in 2021 (early timeline). Comparing the lung involvements in the vaccinated group who received different vaccine types, no significant differences were noted (*p* = 0.46) (see Tables 8 and 9).

**TABLE 8:** Effects of vaccination type on the lung involvements.

Vaccine name	Vaccinated patients	Lung involvement (mean rank)*	*p*
Sinopharm	257	8.16 ± 6.11	0.46
AstraZeneca	22	7.16 ± 7.14	
Barekat	19	8.05 ± 5.94	
Others	36	9.61 ± 5.43	

* Lung involvement scores, ranging 0 to 25.

**TABLE 9:** The effect of difference in hospital admission dates on lung involvements.

	Vaccinated			Non‐vaccinated		
Hospital admission date	Patients number	Lung involvement (mean ± SD)*	***p***	Patients number	Pulmonary involvement (mean ± SD)	***p***
March 21 to Sept. 22, 2021	69	6.26 ± 11.14	0.001	13.25 ± 5.54	110	0.02
Sept. 23, 2021 to Mar. 20, 2022	201	5.78 ± 8.81		7.17 ± 12.55	87	
Mar. 21 to Sept. 22, 2022	64	3.69 ± 3.33		7.15 ± 8.44	16	

* Lung involvement scores, ranging 0 to 25.

Lastly, the study findings indicated that the severity of the patients' lung involvements significantly and inversely correlated with the frequency of vaccinations. The patients who received the COVID‐19 vaccine twice developed significantly less lung involvements (*n* = 169.98) compared with those who received only one vaccine dose (*n* = 197.31) (*p* = 0.03). Also, the severity of the patients' lung involvements was significantly less (*n* = 119.02) than that of those who received only one vaccine dose (*n* = 169.98) (*p* = 0.0001) (see Table 10).

**TABLE 10:** Effects of vaccination dosage on the lung involvements.

Vaccination dose	Patients number	Lung involvement (mean rank)*	*p*
One dose	89	197.31	< 0.0001
Two doses	181	169.98	
Three doses	64	119.02	

* Lung involvement scores, ranging 0 to 25.

## 4 Discussion

This retrospective study was conducted on a total of 547 patients' datasets with COVID‐19 infection to evaluate the efficacy of COVID‐19 vaccination, its contribution to the severity of lung involvements, and the mortality rates among a sample of Iranian subjects. Before admission, patients received symptomatic treatment, and during hospital stay, the standard WHO treatment, based on the disease severity, was administered as reflected in Table 1. The data from the lung CT images primarily suggest that vaccination against COVID‐19 infection significantly lowers the severity of the lung involvements while improving the blood oxygen saturation levels in a dose‐dependent manner in adult Iranians of either gender regardless of age.

Although the focus of this study was on COVID‐19 pneumonia and the disease severity based on the examination of lung CT images, several earlier studies have evaluated the vaccination impact on the clinical outcomes in patients with COVID‐19 pneumonia [32, 33, 34, 35]. For instance, based on the findings of a former study by Jamaati et al., the number of repeated Omicron vaccinations correlated well with better clinical prognosis [36]. Also, in another study, Grapsa et al. have demonstrated that full COVID‐19 vaccination can significantly reduce the duration of ICU stay and mortality rate, even in patients with acute respiratory distress syndrome [32]. A meta‐analysis has reported the efficacy of the COVID‐19 vaccination to be between 86.1% and 95.3% [33] depending on the number of repeated vaccinations. However, the latter study has reported a significantly less mortality in patients with COVID‐19 pneumonia if they were vaccinated more than once [34].

In terms of epidemiological priority, a major suggestion by former studies and consistent with the US Center for Disease Control and Prevention (CDC) is that COVID‐19 vaccination should be given a higher priority to individuals over the age of 60 because it markedly lowers their hospital stay and mortality rates [35, 37, 38]. In a former retrospective study on patients with COVID‐19 pneumonia [15], the mean score for the lung lesions assigned to CT scan images was significantly lower if they were younger than 60 years old. This study attributed the low mean score to the impact of multiple vaccinations. After the outbreak of the COVID‐19 delta variant, the efficacy of several vaccines against COVID‐19, such as BNT162b2, mRNA‐1273, and Ad26COV2.3, was evaluated by another study [39]. Based on the findings, the vaccines' efficacy was reduced after the delta variant emerged. However, its efficacy with respect to hospital stay did not change in individuals older than 65 years old who had received both the BNT162b2 and mRNA‐1273 vaccines [39].

The protective effect of the second or additional vaccinations usually emerges after approximately 2 weeks. Although vaccination is the most efficient tool to fight against the COVID‐19 pandemic, it does not necessarily lead to 100% protection. Consequently, the individuals' response depends on the integrity of their immune system. Thus, further clinical trials should be conducted to better understand how multiple factors can influence the vaccines' immunogenicity, such as viral mutations, age and senility, and the existence of immuno‐compromised conditions [40].

It is known that differences exist in humans based on gender to fight against COVID‐19 infection and initiating the subsequent inflammatory responses. In this context, a major determinant is the presence of steroidal and gender‐related hormones in women that make them less susceptible to viral infections [41]. Nevertheless, the results of the current study suggest that vaccination in both males and females can lead to significant reductions in the severity of the lung involvements due to COVID‐19 infection. With respect to multiple vaccinations, our retrospective study data suggest that the lung involvements become significantly less severe after a second or third vaccine booster. Based on the results of a recent meta‐analysis, COVID‐19 vaccine efficacy against severe COVID‐19 infection remained high, although it diminished somewhat by 6 months after full vaccination. By contrast, the vaccine efficacy against the symptomatic disease decreased by approximately 20–30% within 6 months [42].

The above findings are in contrast with those reported by an earlier study conducted in 2021 by Rzamski et al. on 91 hospitalized patients with COVID‐19 infection who had received multiple vaccinations against COVID‐19 virus [43]. However, there were no significant differences among the patients with respect to the severity of lung involvements, fever intensity, and blood oxygen saturations. That study and a more recent one conducted in vaccinated versus non‐vaccinated patients have suggested that severe lung involvements and high mortality rates do not commonly occur in people who have received COVID‐19 vaccinations more than once [37, 43].

Another point that should be considered is that in comparison of clinical findings between two groups of vaccinated and non‐vaccinated, we did not find any significant difference in the frequency of mentioned symptoms (Table 5). However, the severity of symptoms was not assessed in our study, which might be different among the two groups.

In the current study, the mean age of vaccinated patients (62 years) was significantly higher than that of the non‐vaccinated group (52 years). This is likely due to the priority that older individuals received for vaccination based on the established WHO guidelines. Therefore, age might have had a confounding effect on results. It is also likely that old age is independently linked to better clinical outcomes and interventions, for example, intubation. To omit this confounding factor, we analyzed the vaccination impact on mortality and intubation rates among patients older than 65 years. The results indicated significant decreases in the mortality (*p* = 0.007) and intubation (*p* = 0.03) rates among the vaccinated versus non‐vaccinated patients in this age group. The insignificant decrease in the ICU admissions in the vaccinated group versus non‐vaccinated groups, as reflected in Table 2, is consistent with previous studies results [37]. Also, it should be mentioned that considering risk factors for ICU admission, we excluded patients with known diabetes mellitus, heart failure, and underlying parenchymal lung diseases. However, patients with several other risk factors such as age > 65 years, respiratory rate > 25, and elevated CRP [44] were not excluded and might have been affected by confounding factors.

Given the reduced severity of the COVID‐19 infection reported in various jurisdictions, there are legitimate concerns about the frequent injection of boosters in immuno‐compromised patients. This issue raises the concern whether the vaccine boosters may cause more harm than good to these people [45]. Considering two earlier reports, suggesting that the immune system may be weakened after two COVID‐19 vaccinations, a third booster became popular to enhance immunity against this infection in various countries [46, 47].

The above strategy was later supported by Lytras et al. who found that the mRNA vaccine efficacy against COVID‐19 infection was progressively reduced by 5% per month following the initial vaccination [48]. However, the first and second administrations of mRNA vaccines of BNT162b2, mRNA‐1273, and ChAdOxl variants have still provided optimal efficacy and antibody response against COVID‐19 infection. These vaccines have been able to reduce the subsequent hospital stays and mortality rates in various populations [49, 50]. In this context, additional boosters remain a promising idea to enhance the immune system in individuals older than 75 years and in those with serious comorbidities [34]. Based on available evidence, vaccination of the vast majority of people against COVID‐19 should be carried out at least twice so adequate immunity is developed in average persons, while the side effects and mortality are reduced considerably [51].

One important issue is considering differences among the virus variants, which result in variable disease severity. We compared the disease severity across three timelines based on the dates of hospital admissions. Our notable finding was that the patients' disease severity was considerably less over later timelines (from late 2021 to Sept. 2022) compared with that of the early timelines (Mar.–Sept. 2021). It is likely that the differences in the disease severity could be due to differences in the virus variants and not solely because of the vaccination boosters.

### 4.1 Final Remarks

Considering the above facts, public health authorities and policy makers are advised to keep up with the new strategies on public vaccination initiatives, with the aim being the best possible health outcomes for the citizens [52]. Educating and encouraging the general public to take all steps to safeguard their health should be the ultimate goal if the health care system desires to win the people's trust [53]. Despite the rapid development of numerous vaccines against COVID‐19 infection worldwide, the emergence of new species of the virus has led to faster progress in producing better vaccines. However, much effort remains to be made before this epidemic is brought under full control [5]. We look forward to witnessing ample coordinated efforts to improve the efficacy of the vaccines for various populations in different ages and genders [54].

## 5 Conclusions

The results of the current study provided convincing evidence that vaccination against COVID‐19 infection lowers the severity of lung involvements while improving the blood oxygen saturation in patients with COVID‐19 pneumonia. The study data further indicated that the vaccination significantly decreased mortality and intubation rates among patients older than 65 years old, but it may not have significant effects among other patients' groups. However, the results provided ample evidence that appropriate approaches must be taken to raise public awareness and attract their acceptance of the benefits of vaccination. Such a strategy shall undoubtedly lead to significant reductions of the infection's side effects in Iranian society as well.

## Author Contributions

B.O. and E.A. performed data analysis, generated reports of patients' CT scans, and wrote the manuscript. M.T. collected the data and relevant information, patient symptoms, and clinical histories. S.S. served as the scientific advisor of the manuscript. A.M. performed data analysis.

## Ethics Statement

The study protocol was approved by the Arak University of Medical Sciences' Committee on Research Ethics (Approval code: IR.ARAKMU.REC.1401.183). All procedures and protocols followed in this study were reviewed and approved by the committee, ensuring that they complied with the Helsinki Declaration (1964) and its newer amendments.

## Consent

Written informed consent had been obtained from the patients at the time of admission to the hospital allowing the institution to publish reports generated from the retrospective data on record and consistent with this journal's patient consent policy.

## Conflicts of Interest

The authors declare no conflicts of interest.

## Data Availability

The data that support the findings of this study are available on request from the corresponding author.
